# A National Study on the Effects of Concussion in Collegiate Athletes and US Military Service Academy Members: The NCAA–DoD Concussion Assessment, Research and Education (CARE) Consortium Structure and Methods

**DOI:** 10.1007/s40279-017-0707-1

**Published:** 2017-03-09

**Authors:** Steven P. Broglio, Michael McCrea, Thomas McAllister, Jaroslaw Harezlak, Barry Katz, Dallas Hack, Brian Hainline, CARE Consortium Investigators, April Hoy, Joseph B. Hazzard, Louise A. Kelly, Justus D. Ortega, Nicholas Port, Margot Putukian, T. Dianne Langford, Ryan Tierney, Darren E. Campbell, Gerald McGinty, Patrick O’Donnell, Holly J. Benjamin, Thomas Buckley, Thomas W. Kaminski, James R. Clugston, Julianne D. Schmidt, Luis A. Feigenbaum, James T. Eckner, Kevin Guskiewicz, Jason P. Mihalik, Jessica Dysart Miles, Christina L. Master, Micky Collins, Anthony P. Kontos, Jeffrey J. Bazarian, Sara P. D. Chrisman, Christopher Todd Bullers, Christopher M. Miles, Brian H. Dykhuizen

**Affiliations:** 1 NeuroTrauma Research Laboratory, University of Michigan Injury Center, University of Michigan, 401 Washtenaw Ave, Ann Arbor, MI 48109 USA; 2 Departments of Neurosurgery and Neurology, Medical College of Wisconsin, Milwaukee, WI USA; 3 Department of Psychiatry, Indiana University School of Medicine, Indianapolis, IN USA; 4 Department of Epidemiology and Biostatistics, Indiana University, Bloomington, IN USA; 5 Department of Biostatistics, Indiana University, Indianapolis, IN USA; 6 National Collegiate Athletic Association, Indianapolis, IN USA

## Abstract

**Background:**

The natural history of mild traumatic brain injury (TBI) or concussion remains poorly defined and no objective biomarker of physiological recovery exists for clinical use. The National Collegiate Athletic Association (NCAA) and the US Department of Defense (DoD) established the Concussion Assessment, Research and Education (CARE) Consortium to study the natural history of clinical and neurobiological recovery after concussion in the service of improved injury prevention, safety and medical care for student-athletes and military personnel.

**Objectives:**

The objectives of this paper were to (i) describe the background and driving rationale for the CARE Consortium; (ii) outline the infrastructure of the Consortium policies, procedures, and governance; (iii) describe the longitudinal 6-month clinical and neurobiological study methodology; and (iv) characterize special considerations in the design and implementation of a multicenter trial.

**Methods:**

Beginning Fall 2014, CARE Consortium institutions have recruited and enrolled 23,533 student-athletes and military service academy students (approximately 90% of eligible student-athletes and cadets; 64.6% male, 35.4% female). A total of 1174 concussions have been diagnosed in participating subjects, with both concussion and baseline cases deposited in the Federal Interagency Traumatic Brain Injury Research (FITBIR) database.

**Conclusions:**

Challenges have included coordinating regulatory issues across civilian and military institutions, operationalizing study procedures, neuroimaging protocol harmonization across sites and platforms, construction and maintenance of a relational database, and data quality and integrity monitoring. The NCAA–DoD CARE Consortium represents a comprehensive investigation of concussion in student-athletes and military service academy students. The richly characterized study sample and multidimensional approach provide an opportunity to advance the field of concussion science, not only among student athletes but in all populations at risk for mild TBI.

## Key Points

**Table:** 

Despite similar injury mechanisms and rates among military cadets and women, concussion research to date has largely focused on male athletes participating in contact and collision sports.
The National Collegiate Athletic Association and Department of Defense established the Concussion Assessment, Research and Education (CARE) Consortium to study the natural history of clinical and neurobiological recovery following concussion in cadets and athletes from all sports.
The CARE Consortium is a 30-site investigation enrolling approximately 25,000 military service academy cadets and university student athletes, and capturing 1200 concussions.

## Introduction

Concussion (mild traumatic brain injury [TBI]) is a fundamental concern facing the US military, the sports medicine community, and society at large. Over the past 20 years, sports-related concussion (SRC) has become increasingly recognized as a major public health issue in the US and worldwide, with growing focus and concern among clinicians, researchers, sporting organizations, and student athletes [1–5]. In some instances, the clinical effects of concussion are subtle and are difficult to detect with current assessment tools [6, 7]. In addition, student athletes may underreport their symptoms and falsely inflate their level of recovery, either because of competing messages from stakeholders [8] or in the hope of a rapid return to competition [9, 10]. Moreover, ‘signal detection’ on clinical measures (e.g. cognitive testing) typically diminishes quickly in the acute phase [6], suggesting that the standard clinical assessment of concussion may represent a surrogate index of recovery, not a direct measure of brain structure and function after concussion [11]. Collectively, these issues make the clinical management of concussion a challenge, not only in sports medicine but in the setting of military combat casualty care and civilian trauma.

Several large prospective studies have attempted to clarify the typical time course of *clinical* recovery in symptoms, cognitive functioning, and postural stability after concussion [7, 12–14]; however, the vast majority of these studies have focused on men, predominantly in the contact/collision sports of American football and ice hockey. Considerably less data are available on the natural history of concussion in female student athletes, sports other than football and ice hockey, or military service academy students. In addition, critical questions remain about the acute neurobiological effects of concussion on brain structure and function. Most concerning is that a window of cerebral vulnerability may extend beyond the point of clinical recovery, leaving the brain physiologically compromised and student athletes at heightened risk of repetitive injury [14].

Beyond the acute effects of a concussion, repetitive injuries are known to be associated with subsequent injury [15], and may be associated with slower recovery after an injury event [15] and chronically-elevated post-concussive symptoms [16]. In addition, there are growing concerns that repetitive concussion and/or cumulative head impact exposure through routine participation in contact and collision sports (e.g. football) may be associated with an increased risk of chronic neurologic, neuropsychiatric, and neurobehavioral problems [17–20], including the possibility of chronic traumatic encephalopathy (CTE) [21–24]. While preliminary findings suggest a correlational association between repetitive head impacts and these outcomes [22, 25], a clear causal relationship has not been established [26], leaving a significant gap in our understanding of the natural history of concussion and repetitive head impact exposure.

**Fig. 1:**
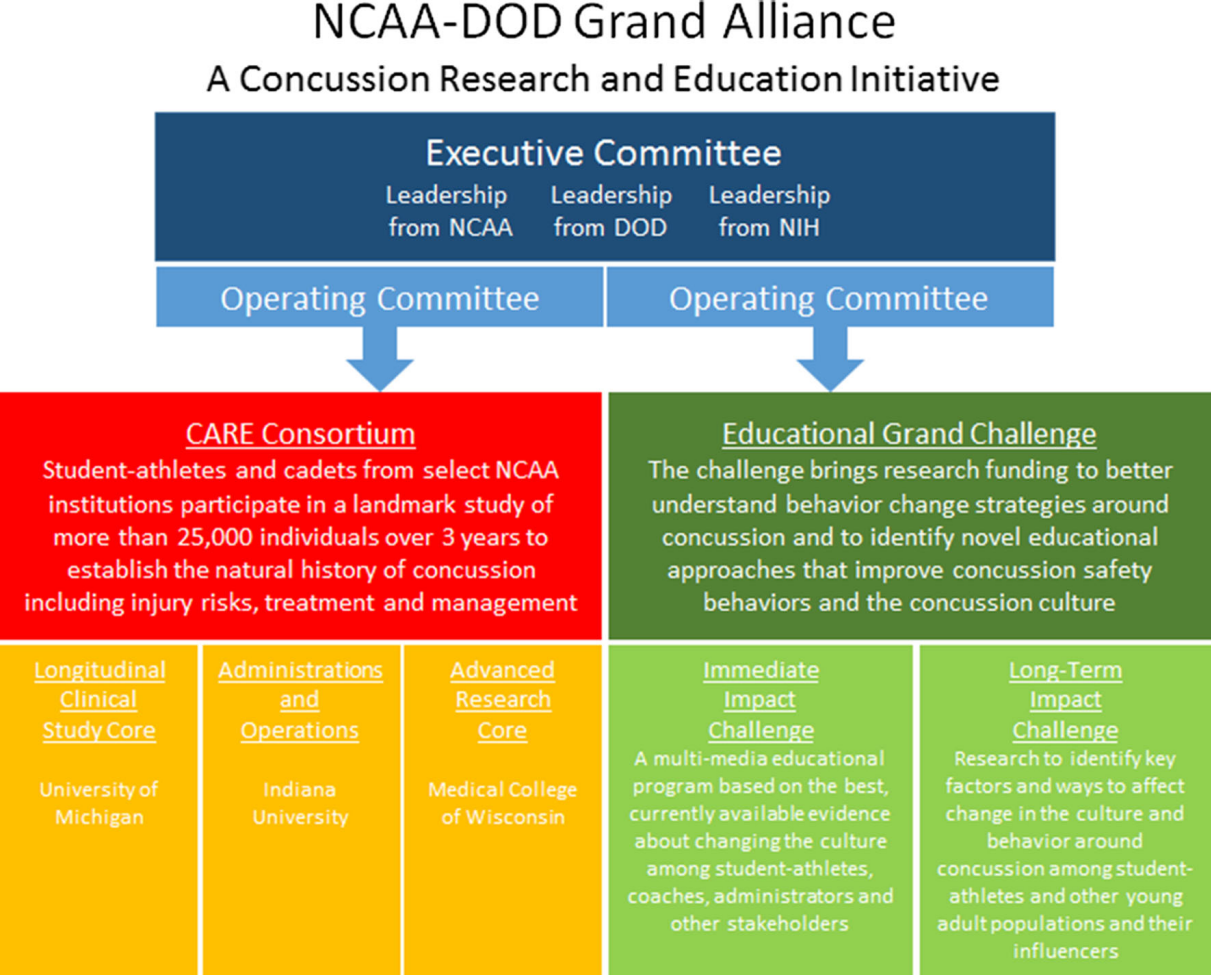
NCAA-DoD Grand Alliance Organizational Structure. *NCAA* National Collegiate Athletic Association, *DoD* Department of Defense, *NIH* National Institutes of Health

In an effort to address these knowledge gaps, the National Collegiate Athletic Association (NCAA) and the US Department of Defense (DoD) established a partnership known as the *Grand Alliance*, with two main arms: the *Educational Grand Challenge* and the *Concussion Research Initiative* (see Fig. 1). The *Educational Grand Challenge* is designed to develop and test strategies to change the culture around concussion in sport, and has been described elsewhere [27]. In parallel, the *Concussion Research Initiative*, supported by US$27.6 million from the NCAA and DoD, is designed to create a concussion research network and to implement studies on the natural history, recovery trajectory, and neurobiology of concussion among college student-athletes and military service academy personnel.

The driving factors underlying this partnership between the NCAA and DoD include (i) recognition of critical gaps in the field of concussion natural history, recovery, and biology; (ii) an awareness of the high incidence and impact of mild TBI/concussion in military service members, military service academy students, and NCAA student athletes; (iii) the need for information about concussion in sports other than American football, and concussion in females; and (iv) the realization that military service members, particularly military service academy students, share similar physical and demographic characteristics with NCAA student athletes. That is, the ‘tactical athlete’ is typically young (18–25 years), healthy, strives for both mental and physical excellence, and sustains concussions under similar mechanisms as service academy personnel. Indeed, nearly 75% of all concussions occurring in active-duty military personnel occur outside of combat [28].

Following a twofold competitive application and review process that included (i) submission of proposals to serve as an independent administrator of a centralized study, and (ii) submission of a clinical study proposal, funds from the United States Army Medical Research Acquisition Activity (USAMRAA), Congressionally Directed Medical Research Program (CDMRP), Combat Casualty Care program, and the NCAA were awarded in the Fall of 2014 to establish the Concussion Assessment, Research and Education (CARE) Consortium as the scientific and operational framework for the Concussion Research Initiative of the NCAA–DoD Grand Alliance. Herein, we describe the key elements of the CARE Consortium structure and function, the methodology of the clinical and neurobiological study cores, and the strengths and limitations of a large multisite study approach to addressing important questions in the field of brain injury science.

## Research Aims and Approach

### Concussion Assessment, Research and Education (CARE) Consortium Structure Overview

**Fig. 2:**
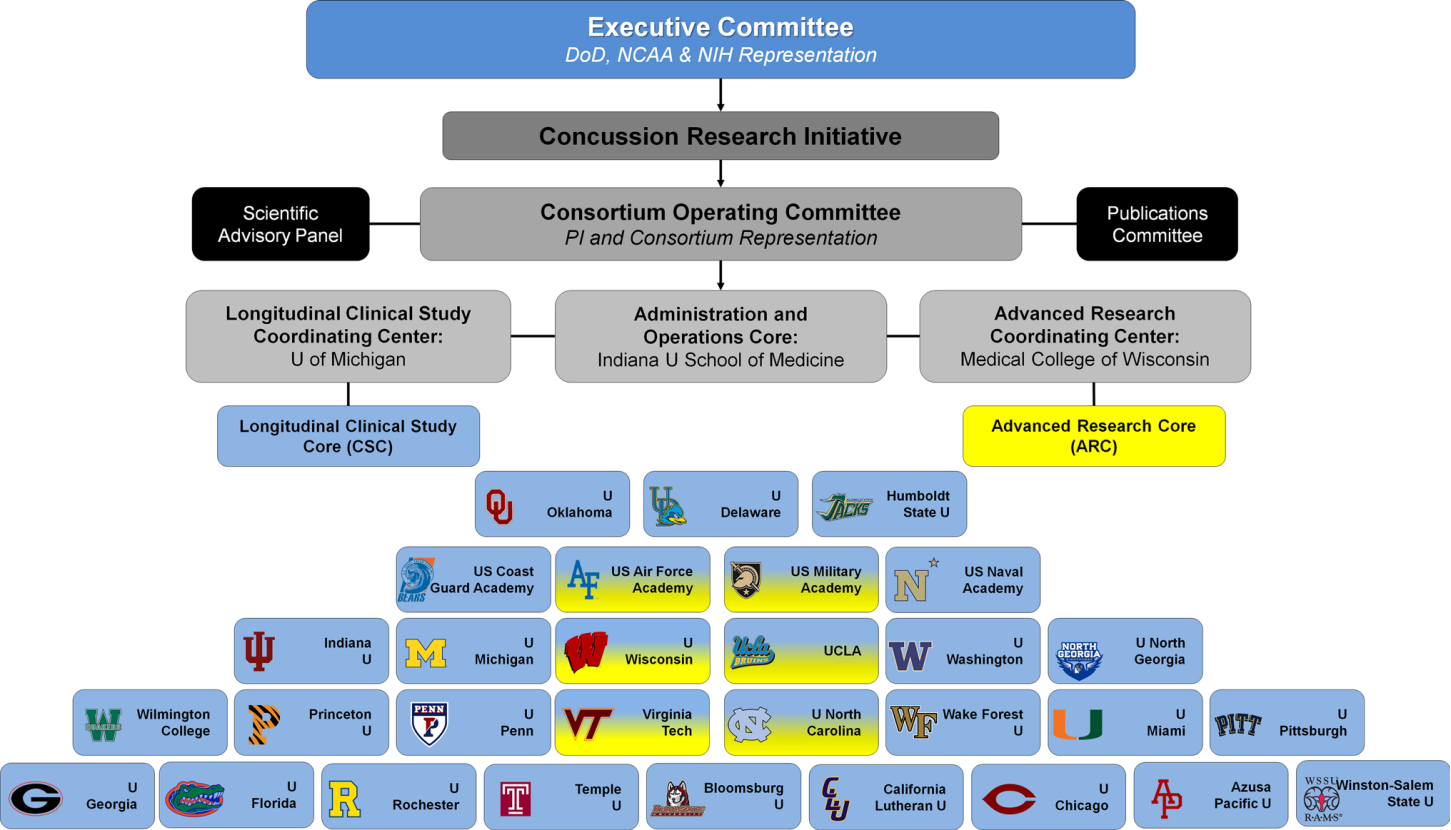
Organizational structure of the CARE Consortium. Year 1 included 15 CSC institutions and 3 ARC institutions; year 2 included 21 CSC institutions and 4 ARC institutions; and year 3 included 30 CSC institutions and 6 ARC institutions. CSC schools are shown in *blue* and ARC schools are shown in *yellow*. Six sites participated in both arms of the study. *NCAA* National Collegiate Athletic Association, *DoD* Department of Defense, *NIH* National Institutes of Health, *CARE* Concussion Assessment, Research, and Education, *PI* principal investigator, *CSC* Clinical Study Core, *ARC* Advanced Research Core

The current CARE Consortium structure is illustrated in Fig. 2. In brief, an Executive Committee consisting of representation from the DoD, NCAA, and National Institutes of Health (NIH) National Institute of Neurological Disorders and Stroke (NINDS) oversee the overall function of the Consortium but have no input into the data collection, analyses, interpretation, or dissemination of any of the Consortium studies. The three CARE principal investigators (PIs) [SB, TM, MM] chair the CARE Consortium Operating Committee, comprised of site PIs of Consortium-participating institutions with expertise in the science and clinical management of concussion. The Operating Committee has representation from a wide array of NCAA conferences and the US Military Service Academies. A Scientific Advisory Board advises the Operating Committee and serves as a resource to the Executive Committee. Composition of the Scientific Advisory Board includes representatives with research and clinical experience in sports and military mild TBI, biostatistics and informatics, and a former NCAA student athlete. An Administrative and Operations Core (AOC), Longitudinal Clinical Study Core (CSC), and Advanced Research Core (ARC) are directed by the three Consortium PIs and their teams at the three lead institutions (Indiana University School of Medicine, University of Michigan, and Medical College of Wisconsin, respectively) and are briefly described below.

### Administration and Operations Core

The AOC functions as the administrative and operations arm of the CARE consortium. The AOC provides Consortium members with oversight and assistance in regulatory and financial functions, maintains the Consortium’s website (http://www.careconsortium.net), standard operating procedures (SOPs), and supports the various Consortium committees (e.g. the Executive Committee, Scientific Advisory Board, Operating Committee, and Publication Committee). Additionally, the AOC houses three support teams to assist in the aims of the CSC and ARC: Biostatistics and Data Management, Bioinformatics, and Biospecimens.

#### Biostatistics and Data Management

The biostatistics and data management activities of the CARE consortium are entrusted to members of the Indiana University Department of Biostatistics and the Datalys Center for Sports Injury Research and Prevention personnel, operating as an integrated unit. The leadership of this core understands the current and future needs of the Consortium, and coordinates roles, responsibilities, and delivery of these needs between the different delivery teams.

The Datalys Center team is the main point of contact to QuesGen Systems (Burlingame, CA, USA), the online data capture portal and data storage server for the Consortium. The Biostatistics and Data Management Team (BDMT) and Datalys collaborated on the development and implementation efforts of the web data capture application in order to ensure the delivery of a robust and user-friendly system with adequate quality-control checks at the point of entry.

Datalys has extensive experience in multisite investigations and is responsible for handling communication with the sites regarding data management issues. This includes communicating data issues from the Indiana University data managers to participating sites, providing user training for the CARE website application, maintaining QuesGen user accounts and data records, and monitoring the ongoing completion of key elements of data entry. The Datalys team also provides tier 1 support to CARE application users, escalating more complex problems to QuesGen.

Indiana University BDMT is responsible for maintaining a high-quality database for the CSC and ARC studies, ensuring that good data management practices are followed throughout the study, and for preparing datasets to support the statistical analysis plan and data-sharing goals of the Consortium. Data managers create programs and procedures to routinely check the database for inconsistencies, identify issues of training, identify trends in data collection, support of data integration efforts, response to ad hoc queries, and preparation of datasets for all the analysis initiatives of the Consortium.

The Biostatisticians on the BDMT are responsible for all of the statistical analyses for the CARE Consortium. They work closely with the CARE Principal Investigators, Indiana University data management, Datalys, and other support teams to identify key questions for data analysis and to translate them into testable statistical hypotheses. The initial steps involve exploratory data analyses, including data visualization, in order to choose the appropriate analytical techniques. Considerations include data distributions, patterns of missing data, possible outliers, and assessment of modeling assumptions (see below). The appropriate statistical models are then identified for the primary analysis of each question. Each step in the analysis is reported to and discussed with the manuscript lead author. At the end of the process, a final statistical report is drafted that includes methods and results, including figures and tables, as well as short introduction and discussion sections.

#### Bioinformatics

The CARE project is generating a rich, multidimensional data set that includes clinical, biomechanical, neuroimaging, genetic, and biomarker variables. As with other similarly complex data sets, a major challenge is how to integrate the information from these various domains in a way that informs our understanding of underlying neurobiological mechanisms. The role of the bioinformatics team is to assist in the analyses, interpretation, and integration of the complex array of data generated by this study across multiple domains, including neuroimaging, genetic, and fluid biomarker data sets.

#### Biospecimens

The biospecimens team is responsible for the oversight and management of biospecimen collection and storage of CARE samples. Protocols developing the acquisition and storage of samples have been developed in collaboration with the ARC. In addition, the biospecimens team has developed training manuals and web-based videos, for use in training sites, in sample collection, processing, storage, and shipping. Moreover, biospecimens team personnel travel to performance sites to provide onsite support in the initiation of the biospecimen collection protocols. Samples are housed in a repository at Indiana University, with extensive procedures in place to ensure the security of the stored samples. Additionally, the biospecimens team regularly interact with the BDMT (also based at Indiana University) to ensure reconciliation and tracking of participant samples.

### Clinical Study Core

Housed at the University of Michigan, the CSC oversees the foundational study on the 6-month natural history of concussion. In the Fall of 2014, the CSC initiated 15 performance sites for data collection, with an additional six schools (21 in total) added in the 2015–2016 academic year, and an additional nine sites (30 in total) added for the 2016–2017 academic year (Fig. 2). Prior to data collection at any site, all site personnel were trained on a standardized protocol for preseason baseline testing and post-injury assessments (described below), and school-level Institutional Review Board (IRB) approval and participant consent were obtained. Protocols approved by the IRB of each performance site also underwent review and approval by the DoD Human Research Protections Office (HRPO).

#### Estimated Enrollment and Concussion Incidence

Estimates of concussion in sport range from as high as 7% annually in contact and collision sports such as football [29] and ice hockey [30] to <1% in non-contact sports [30, 31]. Given the ambitious goals of the Consortium, a large premorbid sample of student-athletes and military service academy students are needed to capture an appropriate number of concussive injuries. To that end, approximately 25,000 unique student-athletes and military service academy students were estimated to be enrolled over the 3-year performance period. This figure was estimated based on an average of 600 eligible student athletes at each performance site and all traditional student-athletes and military service academy students from each of the Service academies (approximately 4000 at each location). Sites were expected to enroll 90% of their eligible student-athletes and/or cadets, and to obtain baseline assessments on 75% of those individuals. Given the breadth of injury incidence, an estimated 768 concussions during the 3-year investigation (year 1–168; year 2–240; year 3–360) were calculated based on an average 2% overall injury rate across all sports and student athletes. To standardize the inclusion of post-injury data, the CARE Consortium adopted the concussion consensus definition produced through evidence-based guidelines [32], which define concussion as “a change in brain function following a force to the head, which may be accompanied by temporary loss of consciousness, but is identified in awake individuals with measures of neurologic and cognitive dysfunction”. Identification, assessment, and diagnosis of concussive events are completed by the research and medical staff at each site.

#### Post-Injury Testing and Management

To best assess clinical differences in concussion outcomes among male/female and contact/non-contact student-athletes and military service academy students, all consenting participants receive an annual pre-season/baseline assessment. Following a concussion diagnosis, assessments are repeated (i) within 6 h of injury; (ii) within 24–48 h; (iii) when the student athlete has been cleared to begin the return-to-play progression; (iv) when the student athlete has been cleared for unrestricted return to play; and (v) 6 months post-concussion (Table 1). Throughout the post-injury process, the performance site’s medical team manages the clinical care of the student athlete based on current standards [1, 3]. In broad terms, student-athletes and military service academy students suspected of sustaining a concussion are immediately removed from play and evaluated by the medical team. If a concussion diagnosis is made, the student athlete is limited or restricted from physical (e.g. team practices/games and conditioning) and cognitive activity (e.g. coursework) until cleared by the medical staff to begin a return-to-play progression [1, 3, 4]. Once the student athlete completes all stages of the progression, he/she is given unrestricted return-to-play clearance.

**Table 1:** CSC and ARC assessment time points and measures

	Pre-season	Acute concussion		Subacute concussion		Post-concussion	
	Baseline	<6 h post-Injury	24–48 h post-injury	Cleared for return-to-play progression	Unrestricted return to play	7 days following return to play^a^	6 months post-injury
Level A	Demographics and medical history; neurocognitive assessment; neurological status; postural stability; symptoms	Neurological status; postural stability; symptoms	Neurocognitive assessment; neurological status; postural stability; symptoms	Neurocognitive assessment; neurological status; postural stability; symptoms	Neurocognitive assessment; neurological status; postural stability; symptoms	Neurocognitive assessment; neurological status; postural stability; symptoms	Neurocognitive assessment; neurological status; postural stability; symptoms
Level B	Advanced postural stability; reaction time; oculomotor; quality of life	Reaction time; oculomotor	Advanced postural stability; reaction time; oculomotor; quality of life	Advanced postural stability; reaction time; oculomotor; quality of life	Advanced postural stability; reaction time; oculomotor; quality of life		Advanced postural stability; reaction time; oculomotor; quality of life
Advanced Research Core Protocol	Genetic testing; blood biomarker collection	Blood biomarker collection	Blood biomarker collection; multimodal MRI	Blood biomarker collection; multimodal MRI		Blood biomarker collection; multimodal MRI	Blood biomarker collection; multimodal MRI

All sites complete all CSC Level A and Level B measures at their discretion. ARC participants complete all CSC Level A measures and the ARC Advanced Protocol

*CSC* Clinical Study Core, *ARC* Advanced Research Core, *MRI* magnetic resonance imaging

^a^Only ARC participants complete Level A testing at the 7 days following the return-to-play time point

#### Assessment Tools

At each assessment time point, all participants receive the Level A assessment battery, considered the minimum level of testing administered at each time point. Selected instruments from Level B, considered as emerging assessments, are added at the discretion of the performance site (Table 1). Time to complete all Level A items is 50–60 min at baseline, 15–20 min at the <6 h point, and 30–40 min at the remaining follow-up times.

Each assessment has been described in detail elsewhere, but, briefly, the Demographics and Personal and Family Medical History unique case report form (CRF) captures demographic data, information on current and previous sport history, concussion, and pre-existing personal and medical history. Neurocognitive functioning is evaluated through a computer and/or pen/paper assessment of psychomotor function, processing speed, attention, learning, memory, and working memory [33]. Given the large number of participating sites and student athletes, sites were allowed to pick their own neurocognitive assessment so performance and predictive ability of several tests could be explored. The ImPACT test is the most widely represented assessment (25 sites), followed by CNS Vital Signs (two sites), the Automated Neuropsychological Assessment Metrics (two sites), and the Cogstate Computerized Cognitive Assessment Tool (one site). The Standardized Assessment of Concussion (SAC) was implemented to assess neurological status [34–37], while the Balance Error Scoring System (BESS) is used to evaluate postural stability [12, 38]. Two measures of self-report symptoms have been implemented, including the Sport Concussion Assessment Tool 3 (SCAT3) symptom list [1] and the Brief Symptom Inventory (BSI)-18 to evaluate depression, anxiety, and somatization [39].

To address emerging instrumentation and methodology for concussion assessment and management, performance sites may also elect to evaluate student athletes on some or all Level B items. The allowable Advanced Postural Stability measures include the NeuroCom Sensory Organization Test [40] and the Sway-Balance smartphone application. A clinical reaction time test may also be used [41], along with the King–Devick Test to evaluate time saccadic eye movements [42], and the Vestibular Ocular Motor Screen (VOMS) to evaluate vestibular and oculomotor function and symptoms [43]. Lastly, the 12-item Short-Form Health Survey (SF-12) [44], Hospital Anxiety and Depression Scale (HADS) [45], and the Satisfaction with Life Scale [46] can be used to evaluate participant quality of life.

### Advanced Research Core (ARC)

The ARC, coordinated by the Medical College of Wisconsin, oversees the CARE foundational study on the neurobiology of concussion. The specific aim of the ARC is to conduct advanced scientific studies that integrate biomechanical, clinical, neuroimaging, neurobiological, and genetic markers of injury to advance our understanding of neurophysiological effects and recovery after concussion in college student-athletes and military service academy students. To complete this aim, a subset of four NCAA institutions (all years) and two military service academies (year 3) participating in the CSC are conducting a more extensive baseline and post-injury protocol incorporating neuroimaging, neurobiological, and/or genetic studies. Like the CSC, the ARC is a prospective, cohort study that includes pre-exposure baseline assessment of a large sample of enrolled student athletes, as well as acute and longitudinal follow-up assessment of a cohort with concussion, a control group of non-concussed student athletes from the same contact sport, and a non-contact-sport student-athlete control group. Table 1 provides an overview of the ARC procedures completed at baseline and each post-injury time point, over and above the clinical testing protocol included in the CSC. Scientifically, our interest in understanding the effects of returning to full participation in unrestricted activities (including exposure to head impact in contact sports) on neurobiological function prompted conduction of ARC procedures at 7 days following return to play rather than on the date of unrestricted return to play.

The ARC calls for enrolling and baseline testing of approximately 3500 student-athletes and cadets, with a detailed post-injury protocol in a projected 500 concussed student athletes, 225 contact-sport student-athlete controls, and 125 non-contact-sport (e.g. baseball, softball, track and field, etc.) controls. The ARC targets specific sports with a known high incidence of concussion, including men’s football, and men’s and women’s ice hockey, lacrosse, soccer, and rugby. The endpoint of concussed student athletes completing the ARC post-injury protocol was based on a conservative rate of concussion in the ARC-targeted sports at the participating sites, taking into account that not all those concussed would be enrolled (i.e. late reports, unreported injuries, failure to meet the study criteria, etc.) and that some degree of attrition would be encountered over the 6-month longitudinal follow-up phase of the study.

In addition to the concussed cohort, the ARC will enroll matched cohorts of contact-sport control student athletes (non-concussed student athletes with a history of head impact exposure from the same contact sport as the concussed student athlete) and non-contact-sport student athletes (non-concussed student athletes from track and field, baseball, etc., without a history of head impact exposure). Both control groups are matched to the concussed group on a combination of demographic, baseline performance, competitive, and exposure variables. The inclusion of these two control groups will enable CARE to differentiate the neurobiological effects of injury versus no injury with exposure (concussed versus contact-sport control) and exposure without injury versus no exposure or injury (contact-sport controls versus non-contact-sport controls).

#### ARC Study Procedures

**Fig. 3:**
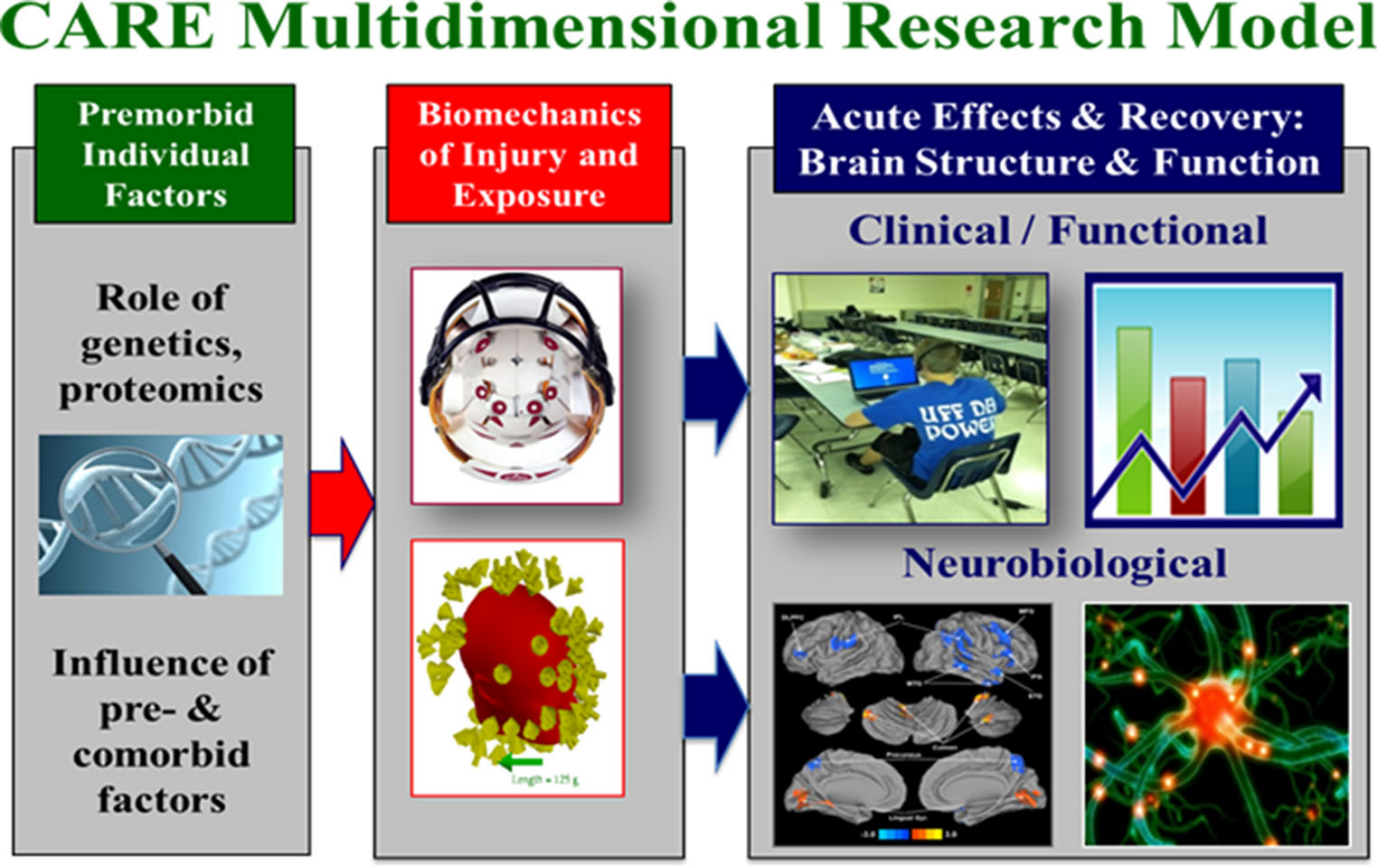
Multidimensional research approach integrating biomechanical, neurobiological, neuroimaging, genetic and clinical data elements of ARC. *CARE* Concussion Assessment, Research, and Education, *ARC* Advanced Research Core

To achieve our specific aims and test our hypotheses associated with the ARC, an extensive, multidimensional set of measures have been implemented to assess concussed and control student athletes across several domains (Fig. 3). Herein, we briefly describe the methods employed as part of the ARC protocols for advanced neuroimaging, fluid biomarkers, genetic testing, and head impact measurement.

#### Advanced Neuroimaging Studies

Concussed student athletes, contact-sport controls, and non-contact-sport controls undergo multimodal magnetic resonance imaging (MRI) on 3.0 Tesla (3T) MRI scanners according to the post-injury timeline (Table 1). While baseline neuroimaging of all participants is recognized as the ‘ideal’ scenario, it was not cost-justified based on the growing literature demonstrating the ability to detect abnormalities in brain structure and function on advanced, multimodal neuroimaging without pre-injury baseline imaging findings [47–52].

The overarching objective of the ARC neuroimaging subcore is to ensure protocols and best practice for quality assurance, data management, and analysis of imaging data. The ARC imaging subcore team is charged with many responsibilities, including (i) obtaining high-quality, multisite data that are consistent over time, and across different MRI systems; (ii) qualifying each scanner on the ARC MRI protocol; (iii) performing appropriate image quality control; (iv) correcting specific classes of image artifacts in each image acquired; (v) monitoring scanner performance longitudinally for structural accuracy and signal stability over the life of the study; and (vi) performing quantitative measurements of all images.

**Fig. 4:**
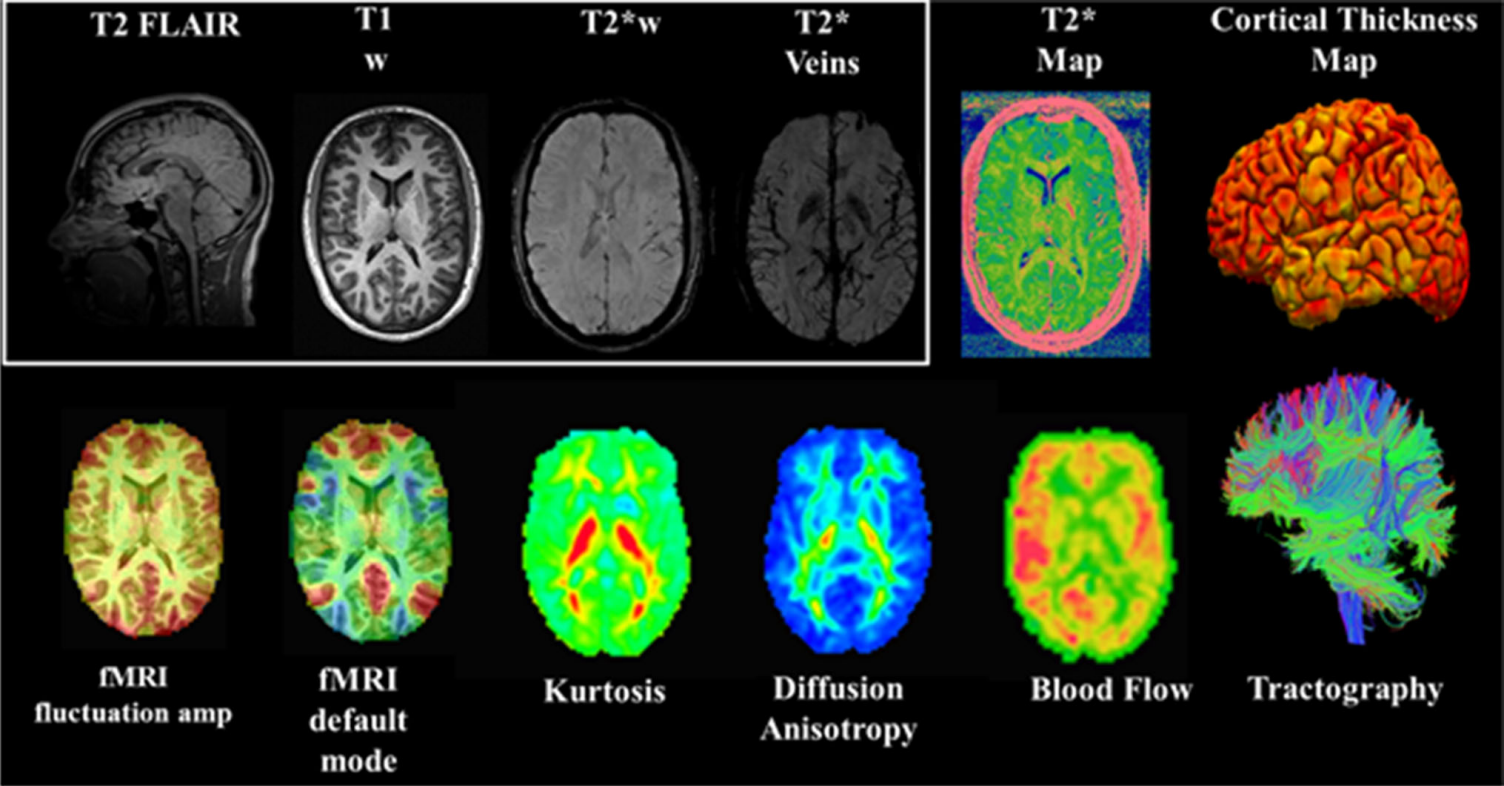
CARE ARC MRI protocol integrating multimodal imaging biomarkers of changes in brain structure and function. *CARE* Concussion Assessment, Research, and Education, *ARC* Advanced Research Core, *MRI* magnetic resonance imaging

The ARC leverages the imaging standardization and quality-control procedures established and validated by the NIH-funded Transforming Research and Clinical Knowledge in Traumatic Brain Injury (TRACK-TBI) study, which will also enable data pooling and comparison across varied mild TBI populations. The ARC has optimized an advanced, multimodal imaging protocol on multiple platforms, including the GE 750, Siemens Trio, Siemens Prisma 3T whole-body scanners, using phase-array head coils, and has cross-validated the protocols across the sites. The data acquisition protocols (Table 2) focus on integrating advances in multimodal imaging technologies specifically well-suited for the study of TBI and concussion (Fig. 4).

**Table 2:** Imaging protocol for the Advanced Research Core

T1-weighted inversion-recovery spoiled gradient-recalled echo (MPRAGE/iSPGR) images for high-spatial resolution of anatomical images. The SPGR images can also be employed for tissue segmentation and volumetric analysis using FreeSurfer, and image registration for functional neural network and diffusion tensor imaging studies
T2-weighted fluid-attenuated inversion recovery (FLAIR) images for pathological detection, such as cerebrovascular disease grading, macroscopic white matter lesion detection and quantification, and microhemorrhage
Diffusion-weighted MRI for detection of microscopic white and gray matter injury using a ‘multishell’ approach (hybrid diffusion imaging) to derive diffusion tensor imaging and diffusion kurtosis imaging markers of injury
T2*-weighted imaging to identify the existence of possible small deposits of blood products associated with microhemorrhage and blood vessel damages
Resting state function MRI (R-fMRI) to study changes in brain functional connectivity
Pseudo-continuous arterial spin labeling (pCASL) to assess alterations in cerebral blood flow

*MRI* magnetic resonance imaging

All ARC sites follow a detailed technical manual outlining the procedures for MRI scanning, data management, and quality control. Imaging data from ARC sites are electronically transferred to the ARC Neuroimaging Repository at the Medical College of Wisconsin for processing, analysis, and secure storage.

The fundamental priorities with respect to analysis of imaging data from the ARC are to (i) validate stability of MRI biomarkers (defined by consistency and reproducibility of findings in controls and human phantoms) across time (within the same scanner) and across sites (across different scanners); (ii) validate diagnostic MRI biomarkers of acute concussive injury; (iii) validate prognostic MRI biomarkers of physiological recovery after concussion; and (iv) correlate MRI biomarkers with clinical outcome measures (symptoms, cognitive testing, balance, etc.) after concussion.

#### Blood Biomarker Studies

Blood samples are collected on all ARC student athletes at the time of baseline testing. Additional biomarkers are then collected within 6 h of injury event, and in conjunction with clinical assessments (symptoms, cognitive, balance) for concussed student athletes, matched contact-sport student-athlete controls, and non-contact-sport student-athlete controls (Table 1). Four acute and two chronic biomarkers tied to TBI are being studied (see Table 3), with additional markers to be considered as the science evolves. The biomarker array selected for the ARC is also closely aligned with the TRACK-TBI study and other NIH-supported investigations of TBI biomarkers. Our primary statistical analysis will determine the sensitivity and specificity of biomarkers alone and in combination to discriminate concussed players from uninjured players as close to the time of injury as possible and throughout the recovery process. Additional analysis will find the best predictive biomarkers of post-concussion recovery.

**Table 3:** Advanced Research Core fluid biomarkers

Acute biomarkers
UCH-L1 (ubiquitin C-terminal hydrolase L1; neuronal protein)
GFAP (glial fibrillary acid protein; astrocytic protein)
SBDP150 (calpain cleaved fragment of alpha II-spectrin breakdown product 150; a neural cell cytoskeleton structural protein)
S100B (S100 calcium-binding protein B; astrocytic protein)
Micro RNA
Chronic biomarkers
MAP-2 (microtubule-associated protein 2; marker of axonal damage)
CNPase (2′,3′-Cyclic-nucleotide 3′-phosphodiesterase; marker of oligodendrocytes)
Micro RNA

#### Genotyping

A portion of the blood samples noted above will be used to generate an aliquot of DNA for the genotyping of >715,000 single nucleotide polymorphisms (SNPs), with a focus on variants having a minor allele frequency ≥5%. Genotyping will be conducted on all student athletes prior to the competitive season and will be analyzed in conjunction with data from clinical assessments (e.g. symptoms, cognitive, balance), head impact sensors, neuroimaging, and blood biomarkers. A targeted genetic approach will be implemented, focusing initially on a small subset of candidate genes that have shown evidence of association in prior TBI-related studies (i.e. apolipoprotein E [APOE], brain-derived neurotrophic factor [BDNF], catechol-O-methyltransferase [COMT], etc.) [53, 54].

#### Head Impact Monitoring

In addition, select student athletes in the ARC were instrumented with head impact measurement technology to assess head impact exposure and concussion biomechanics. Football players in the ARC were outfitted with the Head Impact Telemetry (HIT) System (Simbex, Lebanon, NH, USA) [55]. Head impact data will be correlated with clinical assessments and neuroimaging, blood biomarkers, and genetic testing to build a comprehensive model of injury and recovery.

**Fig. 5:**
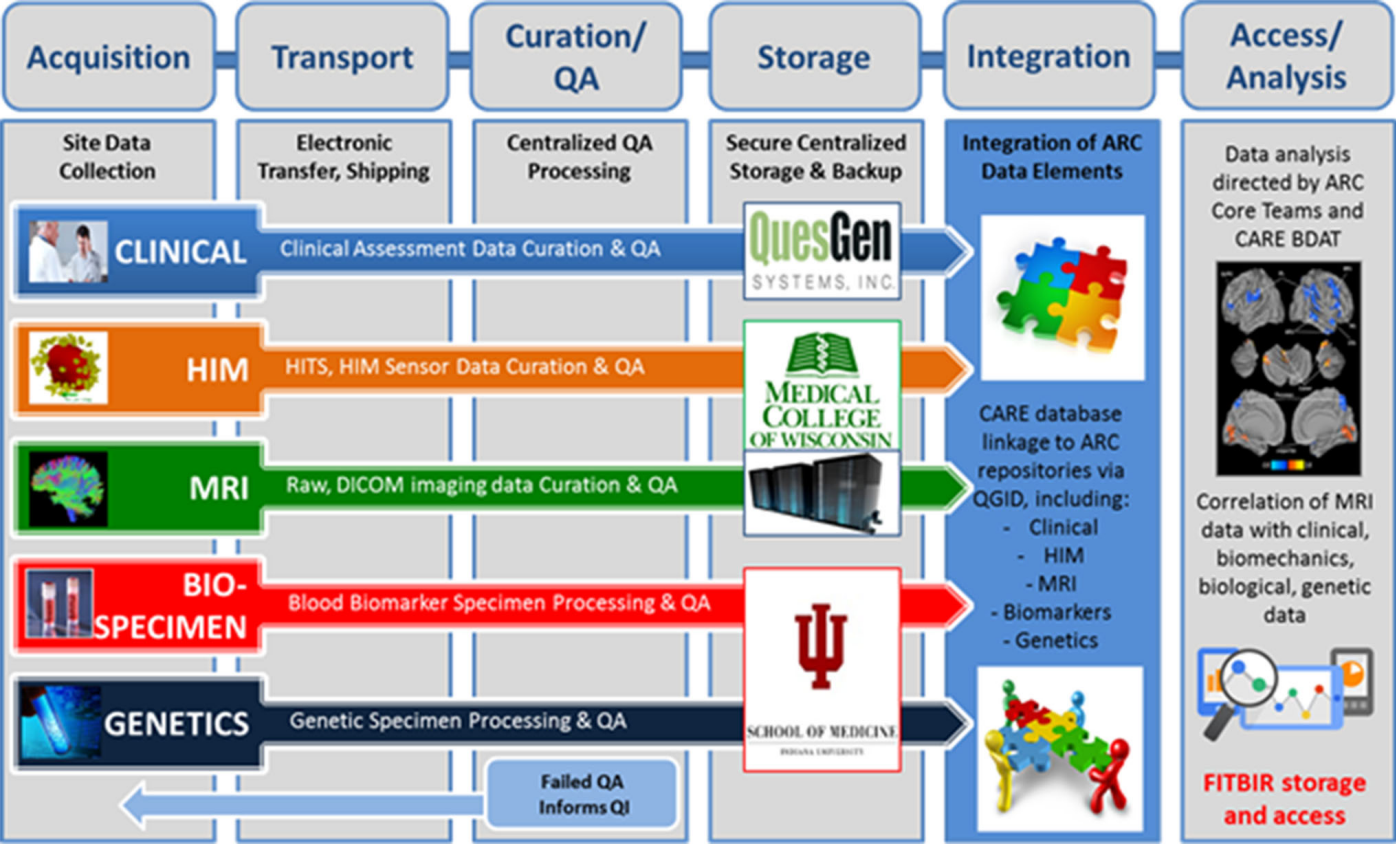
CARE Data Integration Pipelines. *HIM* Head Impact Measurement, *MRI* magnetic resonance imaging, *HITS* Head Impact Telemetry System, *DICOM* Digital Imaging and Communications in Medicine, *QA* quality assurance, *QI* quality improvement, *QGID* Quesgen identification number, *CARE* Concussion Assessment, Research, and Education, *ARC* Advanced Research Core, *FITBIR* Federal Interagency Traumatic Brain Research

Ultimately, the comprehensive nature of the ARC provides a powerful opportunity toward integration of biomechanical, clinical, neuroimaging, neurobiological, and genetic data elements to advance the science on neurophysiological effects and recovery after concussion in college student-athletes and military service academy students. An elaborate plan was required to ensure proper standardization, quality management, and integration of data from each of the ARC subcores, facilitated by the ARC subcore experts and the CARE BDMT (Fig. 5).

## Progress and Challenges To Date

### Human Subjects

As with similar large-scale efforts, attending to regulatory requirements across 30 sites within 3 years, as well as the parent funding agencies, is challenging. Two important strategies facilitated the implementation of this study: development of the template IRB application and informed consent documents, and utilization of an IRB deferral process where possible. The template CSC IRB documents were given initial approval by the University of Michigan IRB, with subsequent approval being issued by the US Army Medical Research and Materiel Command Human Research Protection Office (HRPO), as required by the DoD. The standardized application and consent documents were then distributed to all performance sites for local IRB submission, with only minor modifications allowed based on local IRB policies. The locally approved protocols were also reviewed and approved by the HRPO office. Performance site investigators who wished to conduct similar or concurrent studies obtained free-standing IRB protocol approval for add-on studies that did not interfere with the CARE research. In addition to the standardized forms, HRPO review of both ARC and CSC study documents was further expedited by having a single individual perform all reviews from the Consortium, which maintained review and approval consistency. For those sites also participating in the ARC, site IRBs deferred their review to the Medical College of Wisconsin IRB, and thus only one IRB approval (i.e. the Medical College of Wisconsin) was required to initiate the study. The IRBs of record described above (Indiana University, University of Michigan, Medical College of Wisconsin) operate in accordance with the standards of ethics outlined in the Declaration of Helsinki.

### Enrollment

Launched in Fall 2014, the CARE consortium has completed 24 months of data collection. In the first 24 months of operation, 21 CSC performance sites (including the four ARC sites) have been engaged in data collection and have successfully enrolled 23,533 student-athletes and military service academy students (13,720 male, 9813 female) into the study, well on track to exceed enrollment expectations in both the CSC and ARC. During this time, a total of 1174 concussions have been reported in the CSC, with a post-injury data capture rate of 59% within 6 h of injury; 81.6% at the 24–48 h post-injury time point; 84.1% when the student athlete begins the return-to-play protocol; 79.6% at the return-to-play time point; and 75.6% 6-months post-injury (evaluations between 3 and 9 months post-injury). Similarly, enrollment and injury accrual in the ARC are on track at both the NCAA and MSA ARC sites, with more than 1500 participants enrolled and over 90 concussed student athletes studied under the detailed ARC post-injury protocol. Completeness of the ARC data elements (biomarkers, MRI, genetics) at baseline and the post-injury time points has also been high, despite the intense demands of the protocol.

### Data Collection, Integrity, Storage, and Analysis

To ensure consistency and quality in data collection, each site was provided with a manual of operations providing additional information for every question in the CRFs and detailed instructions on how to best administer each of the assessments outlined above. In addition to this, all site PIs completed in-person training on the assessments with the study PIs, with the requirement that the information would be relayed to their local study teams. Biannual investigator meetings address data collection strategies and concerns at the group level and with individual sites in order to ensure consistent practices across sites and mitigate rater drift and site effects. Lastly, baseline data collection in the first year was completed using pen and paper CRFs which, while effective, had an additional time cost with hand entry into the centralized database. Moving into the second year of the study, the paper CRFs were converted to an electronic format, allowing for direct entry by the study participant. The shift to direct entry has resulted in, overall, 3.9% less missing data. In addition, the electronic data capture allows for range checks, specified response lists and values, and question/response logic that has resulted in overall higher data quality.

To improve the chances of success, data-quality checks have been implemented into the electronic data capture portal. These include a variety of rules and logic checks with additional data scrubbing completed by the BDMT, and with greater detail in preparation for data analysis. In addition, protocol compliance and data integrity is monitored by yearly informed consent documentation reviews and audits to verify that appropriate informed consent has been obtained from our research participants and to ensure that our documentation is being maintained in a compliant manner. Lastly, site visits have been completed to standardize and monitor student-athlete enrollment, data collection, and data storage procedures.

### Neuroimaging Challenges

As in all multicenter neuroimaging studies, CARE has required an intense effort, both centrally within the ARC MRI subcore team of experts and among the ARC site investigators, to ensure maximum standardization, performance, and data quality. Early in the project, the MRI subcore consulted with experts involved in other large-scale, multicenter studies that involved advanced neuroimaging (e.g. ADNI, TRACK-TBI). The collaborative input from these experts was invaluable with respect to sharing of lessons learned in optimizing multimodal imaging protocols across sites and MRI vendor platforms. Our approach toward quality control and integration is designed to closely parallel that of TRACK-TBI, with the goal of eventual cross-comparison of our neuroimaging findings in concussion and TRACK-TBIs in civilian mild TBI.

Additional to developing the specific pulse sequences for our multimodal imaging protocol, the MRI subcore has had to address issues related to MRI scanner upgrades, software updates, head coil functionality and wearability (particularly in large student athletes), and other technical challenges. Furthermore, there was a pressing need to be attentive to the demands on student-athletes and cadets, particularly the total scanning time required at each visit (<1 h). Ultimately, the ARC MRI subcore has followed a philosophy to deploy the most cutting-edge MRI protocol best-suited for the study of concussion, balanced against the technological constraints encountered in optimizing a standardized protocol across multiple sites. Our ability to address new and ongoing issues inherent to multicenter imaging studies in a data-driven and nimble manner has been key to our success.

### Non-Helmeted Sensor Issues

As noted above, one scientific objective of the ARC is to characterize the concussion biomechanics and establish repeated head impact exposure associated with contact- and collision-sport participation. Although some work has been carried out in characterizing the biomechanics of concussion and head impact exposure associated with contact sports such as American football [56–58] and ice hockey [19, 20, 59], little work has been carried out in non-helmeted sports such as soccer because of limitations in currently available technology. Two non-helmet-based options evaluated by the ARC team were determined to have issues that are currently preventing implementation. An alternative mouthguard-based system is currently being evaluated for validity, reliability, and student-athlete ‘usability’. Other non-helmet-based systems are not commercially ready and/or do not have a proper data transfer interface that is required for this study [60].

### Data Storage

Given the anticipated number of total participants, the depth and breadth of data collected, data collection methodologies, and the sensitivity of the data collected, the data storage platform was carefully considered. Indeed, at the time of writing, over 57,709,940 data points had been collected, making a flat data storage platform ineffective. Instead, a relational database maintained by Quesgen was selected. The CARE data capture application is a scalable, browser-independent, web-based application that provides both a researcher interface allowing for paper/pencil data entry from the CRFs, and a participant portal allowing for direct entry of self-reported measures by the study participants. Extensive measures and controls were implemented to ensure security and privacy of the data collected, in addition to accuracy and quality. The application complies with all Federal (i.e. Health Insurance Portability and Accountability [HIPAA]) and Joint Commission on Accreditation of Healthcare Organizations (JCAHO) standards for data access, integrity, confidentiality, auditing, and security.

### Data Analysis

As with any data-centric endeavor of this magnitude, analytic challenges are expected. For example, examination of the baseline data revealed skewed data distributions for many of the instruments, some of which have a large percentage of zero values (e.g. no symptoms) among the student athletes. Thus, statistical models for zero-inflated data are required for valid comparisons among different groups of student athletes. The primary questions of interest will be concerned with post-injury data and the recovery periods for the student athletes who had concussions. Missing data early in the recovery period will be an issue to be dealt with since logistic issues and athlete underreporting often make the <6 h evaluation difficult. Survival analysis methods will be used for time to recovery and will have to account for the correlation among concussions for student athletes with more than one injury. Analysis plans for longitudinal data analysis of assessments measured repeatedly during recovery will also need to take these correlations into account, as well as possible non-linear recovery trajectories. Classical distributional assumptions, especially Gaussianity of these measures noted above, is also suspect. Many of the repeated measures are also observed for different lengths of time for different subjects. Thus, for each analysis we anticipate data issues that will both inform and complicate the choice of statistical model to be used [61].

## Conclusions

In the first 2 years of the investigation, the CARE Consortium has established a national concussion research network and implemented the two initial, planned, foundational studies on the 6-month natural history of concussion and the neurobiology of concussion. Over 23,000 military service academy students and student athletes are enrolled in the study and have undergone baseline testing; nearly 1200 concussions have been identified and studied at multiple time points up to 6 months post-injury. As such, the initiative is positioned to address several key scientific questions surrounding the natural history of SRC and the level of agreement between clinical and physiological recovery. Of particular interest is that 30% of the concussed cohort consists of military service academy students, providing important data on frequency and circumstances of injury in an understudied, at-risk population. In addition, females make up one-third of the current concussed cohort (*n* = 424), making this the largest cohort of female concussion captured to date. Also of note, 21% of the total cohort of concussed participants come from limited or non-contact sports, thus providing important generalizable information on a broader sample of concussion in sport than is currently available.

As with most initiatives of this scope, challenges and limitations are present. Although the enrollment rate overall is quite high, not all student-athletes/military service academy students consent to participate in the studies, thus raising a potential for some bias. We have instituted several measures to ensure consistency of ratings and assessments across participating institutions, yet, as in any study with this number of sites, there is the possibility of site effects and concerns about interrater reliability. In this regard, it is important to consider the reliability of the definition of concussion. There are currently no gold-standard tests such as fluid or imaging biomarkers, cognitive, or other diagnostic tools for concussion, and thus it remains a clinical diagnosis. Although all sites use a common definition and criteria for concussion, concerns remain that subtle differences may exist across sites in diagnostic specificity and sensitivity. In many instances, clinical diagnosis is based on student athlete self-report, sometimes hours or days after the initial event (which can be difficult to identify). This opens the possibility that, on the one hand, concussions are missed due to student athlete underreporting, while, on the other hand, some overdiagnosis may occur in the presence of some non-specific symptoms. Nevertheless, the design of the study reflects current ‘on the field’ realities and state of knowledge, and emphasizes the need for this and other related efforts to improve our understanding of this injury.

Another consideration in interpreting forthcoming data is that subtle, or not so subtle, differences in return-to-play protocols exist across the multiple sites. Even in sites with identical protocols, there is room for clinical interpretation and individual practice can vary. Such differences could impact the injury to return-to-play interval that represents an important outcome measure. However, all CARE participating sites use graded exertion return-to-play protocols, and descriptions of the protocols used at each site are recorded. Furthermore, site effects can be addressed in the statistical modeling of the data, and, in a sample of this size, will be sufficient to adjust for intersite differences.

In summary, the CARE Consortium is on track to make important contributions to our understanding of the natural history, recovery trajectory, and neurobiology of concussion. In doing to, it will serve as a guidepost for changing the clinical care and culture surrounding this injury among student-athletes and military service academy students. It is anticipated that the CARE Consortium will become the building block for both long-term and auxiliary studies.
